# Correlation of left ventricular myocardial work indices with invasive measurement of stroke work in patients with advanced heart failure

**DOI:** 10.3389/fcvm.2022.946540

**Published:** 2022-10-17

**Authors:** Federico Landra, Giulia Elena Mandoli, Benedetta Chiantini, Maria Barilli, Giacomo Merello, Giuseppe De Carli, Carlotta Sciaccaluga, Matteo Lisi, Filippo Flamigni, Flavio D’Ascenzi, Marta Focardi, Massimo Fineschi, Alessandro Iadanza, Sonia Bernazzali, Massimo Maccherini, Serafina Valente, Matteo Cameli

**Affiliations:** ^1^Department of Medical Biotechnologies, Division of Cardiology, University of Siena, Siena, Italy; ^2^Department of Cardiovascular Diseases—Azienda Unitá Sanitaria Locale (AUSL) Romagna, Division of Cardiology, Ospedale Santa Maria delle Croci, Ravenna, Italy; ^3^Cardiology Unit and Laboratorio per le Tecnologie delle Terapie Avanzate (LTTA) Centre, University of Ferrara, Ferrara, Italy; ^4^Interventional Cardiology, Azienda Ospedaliera Universitaria Senese, Policlinico Le Scotte, Siena, Italy; ^5^Department of Cardiac Surgery, University of Siena, Siena, Italy

**Keywords:** myocardial work, stroke work, left ventricle, advanced echocardiography, advanced heart failure, speckle tracking analysis, speckle tracking echocardiography, right heart catheterization

## Abstract

**Objectives:**

This study aimed to explore the correlation between left ventricular (LV) myocardial work (MW) indices and invasively-derived LV stroke work index (SWI) in a cohort of patients with advanced heart failure (AHF) considered for heart transplantation.

**Background:**

Left ventricular MW has emerged as a promising tool for diagnostic and prognostic purposes in heart failure (HF) but its relationship with hemodynamic data derived from right heart catheterization (RHC) has not been assessed in patients with advanced heart failure yet.

**Materials and methods:**

Consecutive patients with AHF considered for heart transplantation from 2016 to 2021 performing RHC and echocardiography as part of the workup were included. Conventional LV functional parameters and LV MW indices, including LV global work index (GWI), LV global constructive work (GCW), LV global wasted work (GWW), LV global work efficiency (GWE), and other were calculated and compared with invasively-measured LV SWI.

**Results:**

The population included 44 patients. Median time between RHC and echocardiography was 0 days (IQR: 0–24). Median age was 60 years (IQR: 54–63). For the most part, etiology of HF was non-ischemic (61.4%) and all patients were either on class NYHA II (61.4%) or III (27.3%). Median left ventricular ejection fraction was 25% (IQR: 22.3–32.3), median NT-proBNP 1,377 pg/ml (IQR: 646–2570). LV global longitudinal strain (GLS) significantly correlated with LV SWI (r = –0.337; *p* = 0.031), whereas, LV ejection fraction (EF) did not (r = 0.308; *p* = 0.050). With regard to LV MW indices, some of them demonstrated correlation with LV SWI, particularly LV GWI (r = 0.425; *p* = 0.006), LV GCW (r = 0.506; *p* = 0.001), LV global positive work (LV GPW; r = 0.464; *p* = 0.003) and LV global systolic constructive work (GSCW; r = 0.471; *p* = 0.002).

**Conclusion:**

Among LV MW indices, LV GCW correlated better with invasively-derived SWI, potentially representing a powerful tool for a more comprehensive evaluation of myocardial function.

## Introduction

The prevalence of people being affected by heart failure (HF) worldwide is incessantly increasing and is now over 60 millions (1). Consensually, the ranks of those in advanced stages of the disease are expanding. Heart transplantation (HTX) is recognized as the most effective destination therapy since median survival time after transplantation exceeds 10 years nowadays (2). In a growing donor organs shortage era, potential receiver patients must undergo a fine selection after comprehensive multi-organ evaluation. Particularly, right heart catheterization (RHC) is routinely performed in order to evaluate pulmonary hemodynamic, since pulmonary hypertension is generally considered a contraindication to HTX.

Right heart catheterization is a tremendously informative exam whose results far exceed a mere pressures measurement. Indeed, RHC provides data regarding flow and resistances, which give additional details about cardiopulmonary physiology. Moreover, measures of stroke work, representative of the area inscribed in the pressure-volume (PV) loop whether of the right or the left ventricle, can be easily derived from other data, bringing deeper insight into myocardial functioning, thus helping the clinician to better characterize the failing heart.

The PV diagram is a well-known tool describing cardiac mechanics and energetics. However fascinating on a theoretical level, its applicability has long remained confined to research setting. A novel echocardiographic method derived from speckle tracking echocardiography (STE) analysis, called “Myocardial Work” (MW), has recently been introduced as a non-invasive derivative of PV curve (3). MW analysis produces a pressure-strain (PS) loop using STE as a surrogate of volume and brachial cuff blood pressure to estimate left ventricular pressures during cardiac cycle.

Myocardial work has already emerged as a promising tool for various pathological conditions (4–16), both for diagnosis and prognosis, but it has only been compared with a completely invasive strategy involving micromanometer and sonomicometry use in animal models to date (3). Therefore, this study aimed to explore the correlation between left ventricular (LV) MW indices and RHC-derived LV stroke work index (SWI) in patients with advanced heart failure considered for heart transplantation.

## Materials and methods

### Patient population

All consecutive patients with advanced heart failure (AHF) considered for HTX from 2016 to 2021 were retrospectively reviewed. Inclusion criteria were: RHC and echocardiography availability, informed consent from the patient. Exclusion criteria were: time between echocardiographic exam and RHC > 3 months, previous left ventricular assist device (LVAD) implantation, previous heart valve surgery/interventions, single chamber ventricular pacing and poor echocardiographic windows. The study was performed in accordance with the Declaration of Helsinki. Local ethical committee approved the study protocol.

### Data collection and standard echocardiography

Patients’ baseline characteristics, vital signs, laboratory tests, medications, echocardiographic data and RHC parameters were retrospectively collected.

All echocardiographic examinations were performed by experienced operators using GE Vivid E80/E95 equipped with an adult 1.5–4.3 MHz phased array transducer, and with an ECG continuously traced, according to the American Society of Echocardiography/European Association of Cardiovascular Imaging recommendations (17, 18).

### Speckle tracking analysis

For STE analysis, endocardial borders and myocardium of all segments from the apical views (four chambers, two chambers, and apical long axis) had to be clearly visualized throughout the whole cardiac circle and ECG track had to be present in each echocardiographic exam. Left ventricular speckle tracking strain is semi-automatically performed by the software in the three apical views and adjusted by the operator, in terms of region of interest (ROI) width and positioning, to optimize endomyocardial tracking. The software warns the operator whether a specific wall segment is not automatically recognized and they may manually adapt ROI and force analysis.

For subsequent MW analysis, markers for aortic, and mitral valves opening and closure are required to set the beginning and the end of each main phase of cardiac cycle (isovolumetric contraction, ejection, isovolumetric relaxation, filling) and they were visually set from the apical long axis view. Moreover, brachial cuff blood pressure are needed to warp in time and amplitude the reference curve for left ventricular pressures estimation and the one detected at the time of echocardiography was used in order to conclude the analysis.

Finally, the software’s output is a series of indices which depict the PS loop from various perspectives (19, 20). In addition, it is provided a graphic representation of the PS loop. The main MW indices are Global Work Index (GWI), which is the total work performed by the heart from mitral valve closure to mitral valve opening, Global Constructive Work (GCW), which is the work performed during shortening in systole adding work during lengthening in isovolumetric relaxation, Global Wasted Work (GWW), which is the work performed during lengthening in systole adding work performed during shortening in isovolumetric relaxation and Global Work Efficiency (GWE), which is constructive work divided by the sum of constructive and wasted work. Additional MW indices are provided: Global Positive Work (GPW), which is the work performed during shortening in systole adding work performed during isovolumetric ventricular contraction, Global Negative Work (GNW), which is the work performed during lengthening in diastole adding work performed during isovolumetric ventricular relaxation, Global Systolic Constructive Work (GSCW), which is the work performed during shortening in systole and Global Systolic Wasted Work (GSWW), which is the work performed during lengthening in systole. MW analysis was performed using EchoPAC software v204 (GE Healthcare).

### Right heart catheterization

Vascular access for RHC examination was obtained with ultrasound guidance through the internal jugular vein under local anesthesia. Adequate zero level was searched using the medium-thoracic line of the supine patient as a reference. Pulmonary artery catheters, also known as Swan-Ganz catheters, were used to measure central venous pressure (CVP), diastolic, and systolic right ventricular pressures, diastolic, systolic, and mean pulmonary artery pressures (PAP) and pulmonary capillary wedge pressure (PCWP). The height of PAP waves was manually measured. Cardiac output was derived by the thermodilution technique (average of five cardiac cycles with < 10% variation) and by the Fick equation. Cardiac index, stroke volume, and stroke volume index were derived indexing cardiac output for body surface area and dividing cardiac output and cardiac index for heart frequency, respectively. Vascular resistances were calculated by the following equations: [(mean pulmonary artery pressure–pulmonary capillary wedge pressure) × 80/cardiac output] for pulmonary vascular resistance and [(mean arterial pressure–right atrial pressure) × 80/cardiac output] for systemic vascular resistance. LV stroke work index (SWI) was retrospectively calculated through the following formula: (mean systemic arterial pressure–mean pulmonary artery wedge pressure) × stroke volume index × 0.0136. All the other parameters were already calculated at the time of catheterization.

### Statistical analysis

Continuous data are presented as mean and standard deviation or as median and interquartile range, as appropriate. Kolmogorov–Smirnov test was used to verify normal distribution of variables. Categorical data are summarized as absolute and relative frequencies. Correlation was calculated using Pearson’s and Spearman’s correlation coefficients, as appropriate. Receiver operating characteristic curves were generated to assess predictive performance of STE-derivate indices for LV SWI. A *p*-value < 0,05 was considered statistically significant. Analysis was performed using SPSS, version 26 (SPSS, Chicago, IL, USA).

## Results

### Patient population

**FIGURE 1:**
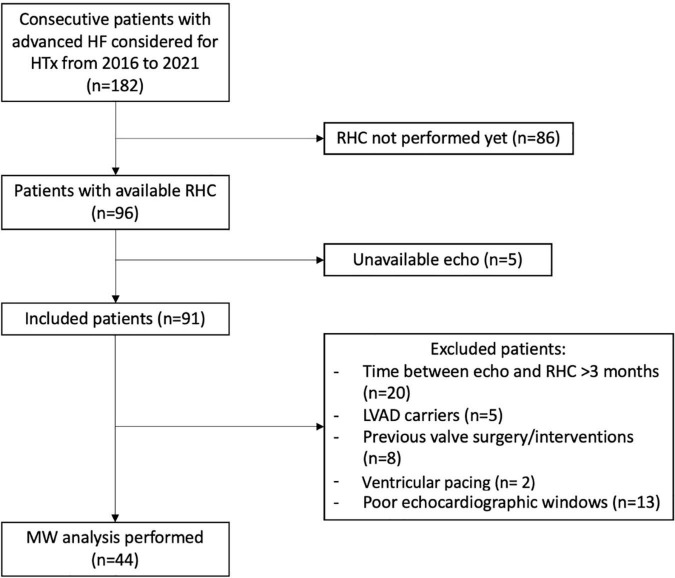
Study flow chart. Inclusion and exclusion criteria as well as patients eventually included in the analysis are reported. HF, heart failure; HTx, heart transplant; RHC, right heart catheterization; LVAD, left ventricular assist device; MW, myocardial work.

One hundred and eighty-two patients with AHF who were evaluated for HTX in our center between 2016 and 2021 were reviewed (*n* = 182). Among them, 86 patients were excluded because they had not performed RHC yet, whereas, five were excluded because of unavailable echo. Moreover, 46 patients were excluded according to exclusion criteria (Figure 1). Therefore, LV MW analysis was performed in 44 patients. Of note, median time between echocardiographic exam and RHC was 0 days (IQR: 0–24).

Median age was 60 years (IQR: 54–62.8) and most of the patients were men (77.3%). Etiology of HF was predominantly non-ischemic (61.4%) and all patients were either on NYHA class II (61.4%) or III (27.3%). HF therapy is described in Table 1. Median NT-proBNP was 1,377 pg/ml (IQR: 646–2,570). For complete baseline characteristics, see Table 1.

**TABLE 1:** Patients characteristics.

Variables	Values
Age (years)	60 (54–63)
Male gender	34 (77%)
BSA (m^2^)	1.96 ± 0.23
Time between RHC and echo (days)	0 (0–24)
Active/former smoker	16 (36%)
Dyslipidemia	28 (64%)
Diabetes	10 (23%)
Hypertension	15 (34%)
Obesity (BMI > 30 kg/mq)	13 (30%)
CKD (eGFR < 60 ml/min/1.73 mq)	6 (14%)
COPD	3 (7%)
Known CAD	12 (27%)
Prior MI	12 (27%)
Prior PCI	9 (21%)
Prior CABG	2 (5%)
Known PAD	7 (16%)
Prior stroke	5 (11%)
Known atrial fibrillation	15 (34%)
**Etiology of HF**	
Ischemic	13 (30%)
Non-ischemic	27 (61%)
**NYHA class**	
I	0 (0%)
II	27 (61%)
III	12 (27%)
IV	0 (0%)
ICD at baseline	38 (86%)
Primary prevention	25 (66%)
Pacemaker	24 (55%)
CRT	17 (71%)
Arrhythmic	3 (7%)
Left bundle branch block	7 (16%)
Any other conduction disturbance	4 (9%)
**Medications**	
Beta-blocker	37 (84%)
ARNI	22 (50%)
MRA	38 (86%)
SGLT2i	5 (11%)
ACEi/ARB	12 (27%)
Ivabradine	4 (9%)
Loop diuretics	33 (75%)
Other diuretics	3 (7%)
Digoxin	10 (23%)
**Laboratory tests**	
Hb (g/dl)	14.2 ± 1.4
PLT (× 10^9^/L)	209 ± 55
WBC (× 10^9^/L)	7.97 ± 2.37
CRP (mg/dl)	0.26 (0.08–0.44)
Creatinine (mg/dl)	1.10 ± 0.23
Na + (mEq/L)	139 ± 3
K + (mEq/L)	4.4 (4.2–4.5)
Bilirubin (mg/dl)	0.65 (0.50–0.78)
GOT (U/L)	21 (18–26)
GPT (U/L)	21 (15–25)
Proteins (g/dl)	7 (7–8)
Glucose (mg/dl)	101 (92–116)
HbA1c (%)	5.9 (5.6–6.4)
BUN (mg/dl)	51 ± 17
Uric acid (mg/dl)	5.5 (4.6–6.8)
Iron (mcg/dl)	95 ± 29
TSH (mlU/L)	2.45 ± 1.46
Total cholesterol (mg/dl)	177 ± 43
HDL-C (mg/dl)	50 ± 14
LDL-C (mg/dl)	90 (73–131)
TG (mg/dl)	146 ± 65
NT-proBNP (pg/ml)	1377 (646–2570)
Troponin (ng/ml)	15.5 (10.8–28.6)
Lactate (mmol/L)	1.12 ± 0.45
LDH (U/L)	196 (174–225)
Mioglobin (mcg/L)	40 (30–46)

BSA, body surface area; RHC, right heart catheterization; BMI, body mass index; CKD, chronic kidney disease; eGFR, estimated glomerular filtration ratio; COPD, chronic obstructive pulmonary disease; CAD, coronary artery disease; MI, myocardial infarction; PCI, percutaneous coronary intervention; CABG, coronary artery bypass graft; PAD, peripheral artery disease; HF, heart failure; ICD, implantable cardioverter defibrillator; CRT, cardiac resynchronization therapy; ARNI, angiotensin receptor neprilysin inhibitor; MRA, mineralocorticoid receptor antagonist; SGLT2i, sodium-glucose co-transporter 2 inhibitor; ACEi, angiotensin converting enzyme inhibitor; ARB, angiotensin receptor blocker; Hb, hemoglobin; PLT, platelets; WBC, white blood cells; CPR, c-reactive protein; GOT, glutamic oxaloacetic transaminase; GPT, glutamic pyruvic transaminase; HbA1c, hemoglobin A1c; BUN, blood urea nitrogen; TSH, thyroid stimulating hormone; HDL-C, high density lipoproteins-cholesterol; LDL-C, low density lipoprotein-cholesterol; TG, triglycerides; NT-proBNP, N terminal pro B type natriuretic peptide; LDH, lactic dehydrogenase.

### Echocardiography

Median left ventricular end-diastolic diameter was 75 mm (IQR: 63–79) and median left ventricular ejection fraction (EF) was 25% (IQR: 22–32). Mean right ventricular mid end-diastolic diameter was 31 ± 4 mm and mean fractional area chance was 39 ± 7%. Diastolic function was impaired, with a median E/e’ of 12 (IQR: 9–16). Median systolic pulmonary artery pressure was 27 mmHg (IQR: 25–44). Only 9.1% of patients had no or mild mitral regurgitation, whereas, only 4.6% of patients had at least moderate aortic regurgitation. Regarding STE parameters, global longitudinal strain (GLS) was impaired, with a median of –5% (IQR: –8—3) [normal range < –19.7% (21)], as well LV MW indices. In particular, mean GWI was 471 ± 294 mmHg% [normal range 1292–2505 mmHg% (22)], median GCW was 612 mmHg% (IQR: 450–932) [normal range 1582–2881 mmHg% (22)], mean GWW was 269 ± 132 mmHg% [normal range 226 ± 28 mmHg% (22)] and median GWE was 67.5% (IQR: 62.0–77.8) [normal range 91 ± 0.8 mmHg% (22)]. For complete echocardiography, parameters see Table 2.

**TABLE 2:** Echocardiographic parameters.

Variables	Values
EDD (mm)	75 (63–79)
EDV (ml)	254 ± 108
RWT	0.25 (0.23–0.29)
LV mass/BSA (g/mq)	160 (131–182)
Stroke volume (ml)	64 ± 26
LV EF (%)	25 (22–32)
LA area (cmq)	28.5 (25.3–33.8)
LA volume (ml)	111.5 (87.8–136.5)
Mid RV EDD (mm)	31 ± 4
TAPSE (mm)	18 (15–20)
RV s’ (m/s)	0.10 (0.09–0.11)
RV FAC (%)	39 ± 8
E (m/s)	0.73 ± 0.26
A (m/s)	0.56 ± 0.26
E/A	1.5 (0.6–2.4)
DT (ms)	158 (124–223)
e’ (m/s)	0.06 (0.04–0.08)
E/e’	12 (9–16)
IVC (mm)	19 ± 4
sPAP (mmHg)	27 (25–44)
**Mitral regurgitation**	
Mild	3 (7%)
Moderate	27 (61%)
Severe	13 (30%)
**Tricuspid regurgitation**	
Mild	14 (32%)
Moderate	16 (36%)
Severe	1 (2%)
**Aortic regurgitation**	
Mild	9 (20%)
Moderate	2 (5%)
Severe	0 (0%)
**Pulmonary regurgitation**	
Mild	24 (55%)
Moderate	5 (11%)
Severe	0 (0%)
Aortic peak velocity (m/s)	1.12 ± 0.24
LV GLS (%)	–5 (–8—3)
LV GWE (%)	67.5 (62.0–77.8)
LV GWI (mmHg%)	471 ± 294
LV GCW (mmHg%)	612 (450–932)
LV GWW (mmHg%)	269 ± 132
LV GPW (mmHg%)	719 ± 310
LV GNW (mmHg%)	256 ± 117
LV GSCW (mmHg%)	653 ± 303
LV GSWW (mmHg%)	206 (147–250)

EDD, end-diastolic diameter; EDV, end-diastolic volume; RWT, relative wall thickness; LV, left ventricle; BSA, body surface area; EF, ejection fraction; LA, left atrium; RV, right ventricle; TAPSE, tricuspid annular plane systolic excursion; FAC, fractional area change; DT, deceleration time; IVC, inferior vena cava; sPAP, systolic pulmonary artery pressure; GLS, global longitudinal strain; GWE, global work efficiency; GWI, global work index; GCW, global constructive work; GWW, global wasted work; GPW, global positive work; GNW, global negative work; GSCW, global systolic constructive work; GSWW, global systolic wasted work.

### Right heart catheterization

Mean right atrial pressure was 7 ± 4 mmHg. Mean pulmonary artery pressure (mPAP) was increased (25 ± 10 mmHg) and median pulmonary capillary wedge pressure (PCWP) was 14 mmHg (IQR: 10–22). Mean vascular pulmonary resistance was 2.46 ± 1.19 mmHg/l/min. Mean LV SWI was 29.84 ± 9.51 mmHg*ml/m^2^. RHC parameters are shown in Table 3.

**TABLE 3:** Right heart catheterization parameters.

Variables	Values
Mean right atrial pressure (mmHg)	7 ± 4
sPAP (mmHg)	38 ± 14
dPAP (mmHg)	15 (11–24)
mPAP (mmHg)	25 ± 10
PCWP (mmHg)	14 (10–22)
Stroke volume index (ml/mq)	32 ± 8
Cardiac index (L/min/mq)	2.14 ± 0.38
Total pulmonary resistance (mmHg/L/min)	6.41 (4.62–8.06)
Vascular pulmonary resistance (mmHg/L/min)	2.46 ± 1.19
Systemic resistance (mmHg/L/min)	19.45 ± 4.79
Systolic aortic pressure (mmHg)	111 ± 13
Diastolic aortic pressure (mmHg)	68 ± 8
Heart rate (bpm)	69 ± 12
LV SWI (mmHg*ml/m^2^)	29.84 ± 9.51

sPAP, systolic pulmonary artery pressure; dPAP, diastolic pulmonary artery pressure; mPAP, mean pulmonary artery pressure; PCWP, pulmonary capillary wedge pressure; LV, left ventricle; SWI, stroke work index.

### Correlation analysis

**FIGURE 2:**
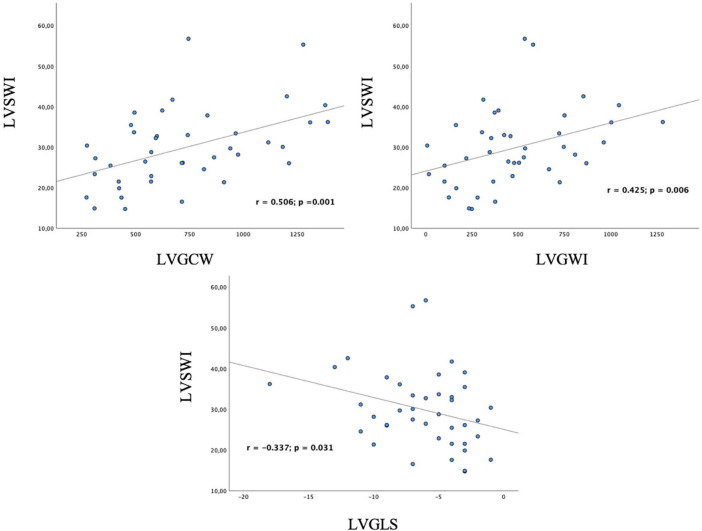
Correlation of left ventricular myocardial work indices and global longitudinal strain with invasive measurement of left ventricular stroke work index. GCW and GWI significantly correlate with invasive measurement of stroke work; GLS significantly correlates as well. LVSWI, left ventricular stroke work index; LVGCW, left ventricular global constructive work; LVGWI, left ventricular global work index; LVGLS, left ventricular global longitudinal strain.

Among traditional parameters of LV systolic function, EF did not significantly correlate with SWI (r = 0.308; *p* = 0.050), whereas, GLS did (r = –0.337; *p* = 0.031). Among MW indices, GWI, GCW, GPW and GSCW significantly correlated with SWI (r = 0.425, *p* = 0.006; r = 0.506, *p* = 0.001; r = 0.464, *p* = 0.003; r = 0.471, *p* = 0.002, respectively). For the complete correlation analysis results, see Table 4; Figure 2. Correlation analysis results between combination of STE-derived indices and left ventricular stroke work index are shown in Table 5. Combination of GCW and GWI showed the best correlation with SWI (r = 0.522, *p* = 0.002).

**TABLE 4:** Correlation analysis results between some echocardiographic indices and left ventricular stroke work index.

LV SWI	Correlation index (r)	*P-value*
LV EF	0.308	0.050
LV GLS	–0.337	**0.031**
LV GWE	0.254	0.110
LV GWI	0.425	**0.006**
LV GCW	0.506	**0.001**
LV GWW	0.076	0.635
LV GPW	0.464	**0.003**
LV GNW	0.183	0.264
LV GSCW	0.471	**0.002**
LV GSWW	0.100	0.545

LV, left ventricle; SWI, stroke work index; EF, ejection fraction; GLS, global longitudinal strain; GWE, global work efficiency; GWI, global work index; GCW, global constructive work; GWW, global wasted work; GPW, global positive work; GNW, global negative work; GSCW, global systolic constructive work; GSWW, global systolic wasted work. Bold values indicate statistical significance.

**TABLE 5:** Correlation analysis results between combination of STE-derived indices and left ventricular stroke work index.

	LVGLS	LVGWI	LVGCW	LVGPW	LVGSCW
LVGLS	/	r = 0.390	r = 0.520	r = 0.505	r = 0.513
		*p* = 0.044	*p* = 0.003	*p* = 0.005	*p* = 0.004
LVGWI	r = 0.390	/	r = 0.522	r = 0.506	r = 0.512
	*p* = 0.044		*p* = 0.002	*p* = 0.005	*p* = 0.004
LVGCW	r = 0.520	r = 0.522	/	r = 0.472	r = 0.473
	*p* = 0.003	*p* = 0.002		*p* = 0.011	*p* = 0.011
LVGPW	r = 0.505	r = 0.506	r = 0.472	/	r = 0.470
	*p* = 0.005	*p* = 0.005	*p* = 0.011		*p* = 0.011
LVGSCW	r = 0.513	r = 0.512	r = 0.473	r = 0.470	/
	*p* = 0.004	*p* = 0.004	*p* = 0.011	*p* = 0.011	

LV, left ventricle; STE, speckle-tracking echocardiography; GLS, global longitudinal strain; GWI, global work index; GCW, global constructive work; GPW, global positive work; GSCW, global systolic constructive work.

### Receiver operating characteristic curves

**FIGURE 3:**
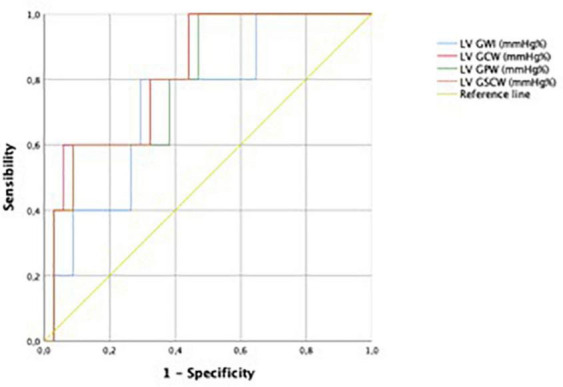
Receiver operating characteristic curves for predictive performance of LVGWI, LVGCW, LVGPW, and LVGSCW for LVSWI. LVGWI, left ventricular global work index; LVGCW, left ventricular global constructive work; LVGPW, left ventricular global positive work; LVGSCW, left ventricular global systolic constructive work; LVSWI, left ventricular stroke work index.

**FIGURE 4:**
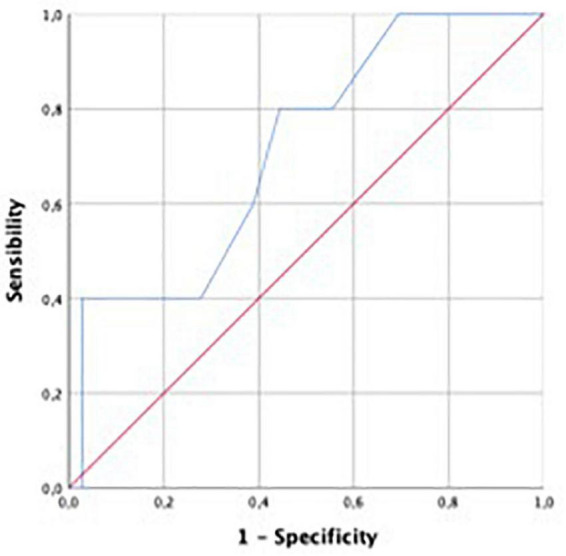
Receiver operating characteristic curve for predictive performance of LVGLS for LVSWI. LVGLS, left ventricular global longitudinal strain; LVSWI, left ventricular stroke work index.

Performance for prediction of LV SWI was greatest for LV GCW (AUC 0.824), followed by LV GCSW (AUC 0.818), LV GPW (AUC 0.800), LV GWI (AUC 0.735), and LV GLS (0.714). For ROC curves see Figures 3, 4.

## Discussion

The main findings of the study can be summarized as follows:

1. Non-invasive measurement of stroke work through myocardial work (MW) calculation significantly correlated with invasively-derived stroke work index in a population of advanced heart failure patients.
2. Among echocardiographic parameters of LV systolic function, correlation with invasive measures increases from classical indices to most recent ones.
3. Ejection fraction is the less reliable tool to evaluate LV systolic function in patients with severely compromised EF, compared to advanced echocardiographic parameters.

The measurement of work performed by the heart has long remained of difficult clinical applicability due to the invasiveness of catheterization. “Myocardial work,” a novel non-invasive echocardiographic method for calculation of work, has already proven its diagnostic and prognostic usefulness in various pathological conditions (5, 8–10, 12, 13). However, it has only been compared with a completely invasive strategy involving micromanometer and sonomicometry use in animal models to date. In fact, in the original paper by Russell et al. (3) the clinical study only compared two groups of patients with invasive vs. non-invasive LV pressure measurements, but both groups involved the use of STE, therefore limiting the comparison to a non-invasive vs. a “hybrid” method. In this study, a correlation analysis between LV MW indices and a completely invasive measurement of work through RHC in patients with advanced heart failure considered for heart transplantation has been performed.

Our results have shown a significant correlation between some indices of myocardial work and invasively-derived stroke work index. More specifically, only the indices, which include the area inscribed in the pressure-strain loop correlated with invasive measurements, that is GWI, GCW, GPW, and GSCW. In fact, each of these indices includes the broader slice of stroke work, namely shortening during the ejection phase. This is not surprising, since invasively calculated work is only stroke work, that is the area inscribed in the pressure-volume loop. Correlation index has reached the highest value with the combination of GWI and GCW, therefore combining two indices could provide more reliable insights into myocardial function. In addition, ROC curves generated to assess predictive performance of significantly correlated STE-derivate indices for LV SWI have shown great areas under the curve, above all GCW, which confirmed itself as the MW index more affine to SWI.

All other myocardial work indices which have not shown a correlation with invasive measurement take into account different aspects of the mechanics and energetics of heart. For instance, GWW and GSWW focus on the work performed by some myocardial segments in the wrong moment of the cardiac cycle, such as lengthening in systole and shortening in diastole. On the contrary, GWE represents an index and for this reason is conceptually different from calculation of work with the invasive method.

However, even though results have shown some degree of correlation between GWI, GCW, GPW, and GSCW with SWI, “r values” are not high enough to support a robust correlation from a statistical point of view. One of the main reasons for which correlation indices were not so high is probably the fact that echocardiographic exams and RHC were not performed on the same day in all cases. It is widely known that these patients suffer from rapid hemodynamic variations indeed.

This study also correlated invasive measurement of work with traditional echocardiographic indices of systolic function, particularly EF and GLS. Even though such indices are conceptually different from work, they also evaluate systolic performance of heart. It is of particular interest the fact that, from EF to MW indices passing through GLS, correlation indices and statistical significance progressively increased. This means that, among the whole range of echocardiographic indices for the evaluation of systolic function available nowadays, newer ones perform better.

Aside from understanding whether these novel indices are reliable or not as compared to invasive measures, one of the key elements toward which future studies should point is how they could be integrated in clinical practice and at what extent they could enhance patients’ management. As a matter of fact, as patients with AHF often need careful clinic visits comprehensive of echocardiogram examination at close time intervals, more sensible advanced parameters of left ventricular systolic function should be implemented to better characterize disease progression and optimize referral for advanced therapies, such as HTX, not to miss the golden window. Since an invasive evaluation strategy with RHC at each time point is inapplicable, a non-invasive evaluation could potentially complement the hemodynamic evaluation at shorter time intervals.

## Limitations

This study was a single-center and retrospective analysis. The main limitations of the study include: the enrolled patients are part of a restricted population of subjects with AHF, which limits the extent of the results to the general population. However, since correlation has been performed between measurements with the two methods in the same individual, this should be of limited concern. Time between echocardiographic exam and right heart catheterization was very low, but not null in all cases. Since these particular patients suffer from rapid hemodynamic variations, even few weeks could be potentially relevant. Nonetheless, if this was true, correlation could only be underestimated. The number of patients in which correlation analysis was performed is relatively low. However, considering the invasive nature of catheterization, this was sufficient for the purpose of the study. If it was not, results would not have reached statistical significance.

## Conclusion

Speckle tracking echocardiography-derived LV MW allows to non-invasively assess the work performed by the heart during a cardiac cycle. This study demonstrated a significant correlation between some MW indices and invasive measurement of LV stroke work derived from RHC in a cohort of patients with AHF considered for heart transplantation, therefore potentially representing a powerful tool for a more comprehensive evaluation of myocardial function.

## Data availability statement

The raw data supporting the conclusions of this article will be made available by the authors, with adequate motivation.

## Ethics statement

The studies involving human participants were reviewed and approved by Comitato Etico Regione Toscana Area Vasta Sud Est. Written informed consent for participation was not required for this study in accordance with the national legislation and the institutional requirements.

## Author contributions

FL, GEM, and MC conceived and designed the study protocol and wrote the manuscript. FL, BC, MB, GM, GD, and CS participated in the acquisition of data. FL performed statistical analysis. ML, FF, FD’A, MFo, MFi, AI, SB, MM, and SV revised the manuscript and participated in the interpretation of results. All authors gave the final approval of the manuscript version to be submitted.

## Abbreviations

LV: left ventricle
MW: myocardial work
HF: heart failure
AHF: advanced heart failure
RHC: right heart catheterization
SWI: stroke work index
GWI: global work index
GCW: global constructive work
GWW: global wasted work
GWE: global work efficiency
GPW: global positive work
GNW: global negative work
GSWC: global systolic constructive work
GSWW: global systolic wasted work
GLS: global longitudinal strain
EF: ejection fraction
HTX: heart transplantation
PV: pressure-volume
STE: speckle tracking echocardiography
PS: pressure-strain
LVAD: left ventricular assist device
ROI: region of interest
CVP: central venous pressure
PAP: pulmonary artery pressure
mPAP: mean pulmonary artery pressure
PCWP: pulmonary capillary wedge pressure.
